# Sustained Effects of Acute Resistance Exercise on Executive Function in Healthy Middle-Aged Adults

**DOI:** 10.3389/fnhum.2021.684848

**Published:** 2021-08-18

**Authors:** Chien-Chih Chou, Ming-Chun Hsueh, Yi-Hsiang Chiu, Wen-Yi Wang, Mei-Yao Huang, Chung-Ju Huang

**Affiliations:** ^1^Graduate Institute of Sport Pedagogy, University of Taipei, Taipei, Taiwan; ^2^Department of Physical Education, Chinese Culture University, Taipei, Taiwan; ^3^Department of Sport Promotion, National Taiwan Sport University, Taoyuan City, Taiwan

**Keywords:** inhibitory control, executive function, resistance exercise, sustained effects, middle-aged adult

## Abstract

The present study examined the sustained effects of acute resistance exercise on inhibitory function in healthy middle-aged adults. Seventy healthy middle-aged adults (mean age = 46.98 ± 5.70 years) were randomly assigned to exercise or control groups, and the Stroop test was administered before, immediately after, and 40 min after exercise. The resistance exercise protocol involved two sets of seven exercises performed for a maximum of 10 repetitions, with 60 s between sets and exercises. Acute resistance exercise resulted in higher Stroop test performance under the incongruent (inhibition) and interference conditions immediately post-exercise and 40 min post-exercise. Furthermore, the difference in scores after 40 min was significant. The findings indicate that a moderately intensive acute resistance exercise could facilitate Stroop performance and has a more beneficial effect on sustaining of cognition that involves executive control at least 40 min.

## Introduction

Cognitive ability is important for daily life as a main component of the health-related quality of life. However, the most impactful change in cognition with increasing age is declining executive function (EF), with cognitive impairments severe enough to impair their everyday functional abilities (Barnes, 2015; Murman, 2015). Encompassing a set of higher-order cognitive processes, EF enables individuals to plan, organize, and complete tasks when engaging in goal-directed actions and adaptive responses to novel or complex situations (Hillman et al., 2012). Age-related decline of EF is the focus of numerous studies on cognitive aging (Salthouse, 2010; Chang et al., 2012b; Lustig and Jantz, 2015; Fjell et al., 2017; Rey-Mermet and Gade, 2018), particularly in inhibitory control among middle-aged adults (Chang and Etnier, 2009b; Chang et al., 2014, 2019), which could be improved via physical activity, such as acute aerobic or resistance exercises (Byun et al., 2014; Chang Y. K. et al., 2017).

According to meta-analyses of exercise and inhibitory control, acute aerobic or resistance exercises have small to moderate effects on inhibitory control in middle-aged populations (Yanagisawa et al., 2010; Chang et al., 2012b; Basso and Suzuki, 2017). Notably, in contrast to aerobic exercise, concentration levels of growth hormone (GH) and insulin-like growth factor-1 (IGF-1) are strongly associated with the degree of resistance exercise intensity (Gregory et al., 2013), and increases in these concentrations are correlated with inhibitory control improvements (Tsai et al., 2014). Furthermore, a systematic review provides compelling evidence for the occurrence of acute resistance exercise-induced increases in inhibitory control performance (Wilke et al., 2019). Inhibitory control refers to the ability to control one’s attention, behavior, thoughts, or emotions to override a strong internal predisposition or external lure while irrelevant information in the interference control and inhibit a prepotent response to allow selection of the appropriate responses (Diamond, 2013). Several studies have observed improvements on inhibitory control after an acute bout of resistance exercise (Chang and Etnier, 2009a; Chang et al., 2014; Johnson et al., 2016; Naderi et al., 2019; Wilke et al., 2019). Although the findings generally support a beneficial immediately effect of acute resistance exercise on cognitive performance (Chang and Etnier, 2009b; Forte et al., 2013; Chang et al., 2014, 2019; Naderi et al., 2019). The few studies that have examined the delayed effects (post-training) on cognitive performance have concluded that the benefits are not maintained (Pontifex et al., 2009; Barella et al., 2010; Chang et al., 2019; Naderi et al., 2019). Thus, whether an acute bout of resistance exercise exerts a sustained effect on inhibition in middle-aged individuals remains to be clarified.

In reviewing the literature on sustained effects of resistance exercise on inhibitory control, Chang and Etnier (2009a) and Chang et al. (2014) both observed facilitation of performance immediately after exercise relative to a control condition on a Stroop task. Furthermore, Naderi et al. (2019) observed that the exercise-induced gains on inhibitory control were larger at 15 min than 180 min after an acute resistance exercise. No such effect was observed for a low-intensity resistance exercise bout, indicating that a moderate-intensity resistance exercise bout was beneficial to inhibitory control. Tsukamoto et al. (2017) observed similar facilitative yet immediate effects of resistance exercise on inhibition relative to a pre-exercise condition. Further support for the beneficial effects of acute exercise on inhibitory control has been garnered from Johnson et al. (2016) who observed facilitation in performance on a Stroop Test after a 30-min recovery period after exercise, but not following a delay of 30 or 60 min. Taken together, these results suggest that changes in inhibitory control could be similar facilitative benefits after 30 min of resistance exercise relative to a pre-exercise condition. These inconsistencies regarding sustained effects expose a lack of clarity regarding the influence of resistance exercise on inhibitory control (Barella et al., 2010; Naderi et al., 2019). Despite these initial inquiries into the immediate benefits of acute resistance exercise for inhibitory aspects of executive control, little research has investigated its sustained effects over time (Barella et al., 2010; Naderi et al., 2019).

Despite these initial inquiries into the effect of acute exercise on inhibitory aspects of executive control, little research has examined this relationship of the time course to the cognitive benefits. Therefore, a rationale is raised for our current theoretical understanding for the acute exercise intensity-cognition interaction based on the arousal-performance interaction theory. A prominent theoretical framework proposed by Davey (1973) revealed that exercise intensity, and its effect on arousal, influences cognitive performance in an inverted-U manner. Specifically, inhibitory control was benefited by moderate-intensity exercise compared to low-intensity exercise (Tsukamoto et al., 2017; Naderi et al., 2019). In addition, the critical indicators of psychophysiology such as insulin-like growth factor, brain-derived neurotrophic factor, fibroblast growth factor 2, and vascular endothelial growth factor (Chang et al., 2012b; Tsai et al., 2014) suggested that moderate-intensity resistance exercise influences cognition performance by optimally elevating arousal.

The purposes of this study was to examine the immediate and sustained effects of acute resistance exercise on inhibition at 5 and 40 min post-exercise after an acute resistance exercise. A previous meta-analysis reported that the largest effects of exercise on cognitive performance could generally be observed during a period of 11–20 min after exercise (Chang et al., 2012a). However, the latter time point was selected based on two lines of evidence. The time point of 5 min immediately post-exercise was selected based on a physiological stimulus for acute increases in GH and IGF-1 levels (Gregory et al., 2013). On other hand, the time point of the 40 min post-exercise was selected due to reductions in state anxiety during processes that influenced cognition. Previous studies have most commonly examined cognition during the initial 17 to 60 min post-exercises to determine acute exercise effects on cognition (Johnson et al., 2016; Tsukamoto et al., 2017). We, therefore, chose 5 min immediate post-exercise as the time point to reflect the more effects of acute exercise on inhibitory control performance. The purpose of this study was to extend the literatures by including immediately post-exercise and 40-min post-exercise that are theoretically and biologically justifiable, and also focusing on changes in inhibitory control performance after an acute resistance exercise. Therefore, we hypothesized that healthy middle-aged adults participating in acute resistance exercise would exhibit immediate improvement in inhibitory control compared with those not participating in the exercise treatment and that effects would be sustained at least 40 min.

## Materials and Methods

### Participants

A total of 70 community-dwelling healthy adults, aged 40–60 years, were initially recruited in Taipei, Taiwan. The potential participants were included if they met the following criteria: (1) they met the requirements of the physical activity readiness questionnaire (PAR-Q), to ensure their safety when performing a single bout of exercise and (2) they achieved a score of more than 26 on the Chinese version of the mini-mental state examination (MMSE), verifying that they may be considered cognitively normal (Guo et al., 1988). The exclusion criteria were as follows: (1) the presence of comorbid conditions, such as autism spectrum disorder, attention deficit hyperactivity disorder, intellectual disability, and serious affective disorder; (2) history of brain injury or neurological disorder; and (3) medical conditions involving the presence of any neurological, psychiatric, musculoskeletal, or cardiovascular problems that would affect exercise ability. After assessment, the participants were randomly assigned to an exercise group (*n* = 35) or a control group (*n* = 35) by drawing lots. Detailed characteristics of the participants are presented in Table 1. The study protocol, informed consent, and all materials were reviewed and approved by a university’s Institutional Review Board in Taipei prior to the experiment.

**TABLE 1:** Summary of the demographic characteristics of the participants.

Variables/Sex/Groups		EG (*n* = 35)	CG (*n* = 35)	Total
Sex (M: F)		15:20	16:19	31:39
Age (years; *M* [SD])	Male	45.97 [5.57]	45.59 [4.24]	
	Female	46.72 [5.51]	49.22 [6.75]	46.98 [5.70]
	Total	46.39 [5.47]	47.56 [5.95]	
Height (cm; *M* [SD])	Male	172.53 [4.61]	172.50 [3.90]	
	Female	160.15 [5.29]	158.32 [3.86]	165.12 [7.98]
	Total	165.46 [7.94]	164.80 [8.12]	
Weight (kg; *M* [SD])	Male	73.95 [5.81]	77.43 [13.79]	
	Female	57.05 [5.88]	53.28 [4.76]	64.30 [13.14]
	Total	64.29 [10.25]	64.32 [15.65]	
Body mass index (kg/m^2^; *M* [SD])	Male	24.85 [1.85]	25.84 [4.12]	
	Female	22.22 [1.70]	21.28 [2.00]	23.36 [3.12]
	Total	23.35 [2.19]	23.37 [3.86]	
Education (years; *M* [SD])	Male	17.20 [2.08]	17.06 [2.62]	
	Female	15.80 [1.82]	15.05 [3.32]	16.19 [2.64]
	Total	16.56 [2.53]	17.06 [2.62]	
Resting HR (bpm)	Male	73.60 [6.49]	72.63 [2.58]	
	Female	71.50 [5.47]	70.37 [4.23]	71.90 [4.94]
	Total	72.40 [5.93]	71.40 [3.70]	
Mini-mental state examination	Male	28.66 [0.82]	28.56 [0.89]	
	Female	28.45 [0.99]	28.47 [0.90]	28.53 [0.90]
	Total	28.54 [0.92]	28.51 [0.87]	

**Resistance exercise**				

Biceps curl-right (lb)	Male	39.07 [6.39]	∼	34.63 [6.16]
	Female	3130 [3.24]	∼	
Biceps curl-left (lb)	Male	35.73 [5.12]	∼	32.77 [5.50]
	Female	30.55 [4.76]	∼	
Back lat pull down (lb)	Male	88.93 [4.38]	∼	79.46 [9.34]
	Female	72.35 [4.22]	∼	
Chest fly (lb)	Male	53.87 [5.42]	∼	40.17 [13.36]
	Female	29.90 [6.23]	∼	
Chest press (lb)	Male	84.67 [9.33]	∼	61.69 [21.84]
	Female	44.45 [7.74]	∼	
Leg curl (lb)	Male	79.47 [10.22]	∼	66.09 [14.02]
	Female	56.05 [5.22]	∼	
Leg press (lb)	Male	175.20 [9.34]	∼	163.26 [13.41]
	Female	154.30 [7.78]	∼	

### Measures

#### Stroop Test

The Stroop test was developed by the Vienna Test System. The Vienna test system is a test system for computerized executive function assessments. With it, digital tests of executive function can be administered and it provides automatic and comprehensive scoring. It includes a series of computerized tasks. Perceptual motor speed is measured when reading color words (e.g., “red,” “yellow,” “blue,” or “green”) and naming the color that the word is (or is not) written in. The test uses correct responses on time as reaction time. The test has 128 trials, with 5 trials for practice. Trials present the four types of Stroop task, namely, the baseline of reading words and naming colors as the congruent condition, and reading and naming words under the incongruent condition; the difference in reaction time between the conditions is the interference tendency and is referred to as the Stroop effect. A positive value indicates an increased interference tendency, whereas a negative value is characteristic of a reduced interference tendency. In the congruent condition, the reading word and naming color stimulus is displayed in the color matching the meaning of the word (e.g., the word “red” is presented in red color). In the incongruent condition, the reading word stimulus is displayed in a color matching the meaning of a different stimulus (e.g., the word “yellow” is displayed in blue color). The participants must read the word (i.e., “yellow”) aloud and disregard the color of the word is written in (i.e., blue). Alternatively, with the naming color stimulus, the participant must state the color of the word (e.g., “red”) and disregard the word’s meaning (e.g., the color green). The stimuli for each condition were displayed on a 15-inch laptop screen, and the test length was approximately 8 to 10 min. The validity and reliability of the Stroop test has been extensively reported (Scarpina and Tagini, 2017; Erdodi et al., 2018). The Stroop test was chosen for the present study based on past evidence that it is both sensitive to the effects of exercise and provides a reliable measure of EF for healthy middle-aged adults (Chang and Etnier, 2009a).

### Exercise Manipulation Check

#### Heart Rate

A Polar watch (Cybex 770T’s CardioTouch, United States) was worn by each participant to measure the participant’s heart rate (HR) during the resistance exercise stage, with the HR data from the HR monitor being recorded at 1-min intervals. HR data were assessed as an indicator of the physiological arousal induced by the resistance exercise. Four HR variables were identified: pre-exercise HR, treatment HR, immediately post-exercise HR, and 40-min post-exercise HR. Pre-exercise HR was determined 60 s before the performance of the first Stroop test (pre-exercise); the treatment HR was the average HR during the moderate intensity or control treatment; and the immediately post-exercise HR and 40-min post-exercise HR were assessed 60 s before the participant performed the Stroop tests for immediately post-exercise and 40 min post-exercise, respectively.

#### Rating of Perceived Exertion

Each participant completed the rating of perceived exertion (RPE), which was defined as their perception of their own level of effort during the exercise. According to the original scale by Borg (1998), the RPE rates a participant’s perceived exertion on a scale of 6 (“very, very light to fairly light exertion”) to 20 (“maximal exertion”). The RPE was recorded at 2-min intervals during the exercise.

### Resistance Exercise Design

The participants were instructed to avoid engaging in any other resistance exercise training or any other physical activities such as jogging, running, yoga, dance, tennis, or table tennis for a week before the experimental session. The 10-repetition maximum (RM) represents the maximum weight an individual can successfully lift in 10 repetitions and approximates 70% of the 1-RM. After stretching, the participants were asked to warm up with light resistance exercise for 10 min, and were then instructed to change the load and to continue the testing process until they reached a load level that they would be able to lift for a maximum of 10 repetitions. Generally, the instructor was able to adjust the loads such that the 10-RM could be measured within four testing sets. The following seven muscle exercises were selected: the bench press, shoulder press, dumbbell rows, alternating bicep curls, triceps pushdowns, leg extensions, and leg curls (American College of Sports Medicine (ACSM), 2013). The load for each of the exercises was determined using the same protocol. The resistance exercise consisted of two sets of 10-RM tests for the seven resistance exercises with 60 s of rest between sets and exercises. The protocol was selected based on previous studies (Chang et al., 2014; Tsai et al., 2014) that have indicated that cognitive performance could be affected positively by using this protocol. HR and RPE, two commonly used indicators for exercise intensity in EF studies, were used to confirm that the participants achieved moderate exercise intensity (Chang et al., 2019).

### Procedures

The participants were requested to visit the Sport Pedagogy Laboratory on two separate testing days at least 48 h apart. Trained laboratory researchers administered questionnaires regarding the inclusion criteria, the 10-RM tests for each of the seven muscle groups, and the Stroop tests. On Day 1, each participant was invited to visit the laboratory at the confirmation stage and was presented with a brief introduction to the experiment. The informed consent form, MMSE, PAR-Q, and medical health history questionnaire were given to them to read and complete. After completing the questionnaires on Day 1, each participant was asked to sit quietly, individually, on a comfortable sofa in a dimly lit room for 15 min and instructed to attach the HR monitor. After the 15-min period, the HR baseline was recorded, and the participant’s 10-RM for each of the muscle groups was determined. The participants were told to avoid the use of stimulants such as caffeine the day before and the day of the tests.

On Day 2, each participant was again asked to sit quietly, individually, on a comfortable sofa in a dimly lit room for 15 min. Then, pretest scores for the Stroop test were collected, and the participant was asked to press the correct color button on the keyboard for each stimulus in each condition. In the exercise groups, the participants then warmed up for 10 min and conducted the resistance exercises for 25–30 min. At the start of each session, the participants were fitted with a Polar Cybex 770T’s CardioTouch HR monitor to assess their physiological arousal and the effects of the selected intensities on cardiovascular responses. Subjective RPE was collected after each set of exercises using a category-interval rating scale ranging from 6 (no exertion at all) to 20 (maximal exertion). In the control group, the participants were instructed to sit quietly in a well-lit room and read exercise-related magazines for 30 min.

Immediately post-exercise (3 to 5 min after the end of the session), we asked each participant to complete the Stroop test again using the same directions and under the same conditions as the pre-exercise test. Afterward, the participant rested for 30 min and then completed all four conditions of the Stroop test again (40-min post-exercise stage). Each of the participants was given US$20 as compensation for participating in the study and was debriefed by a member of the research team.

## Results

### Demographic Analyses

To ensure homogeneity of potential confounders between the control and exercise groups, an analysis of independent samples was applied using a *t*-test or a χ*^2^* test to compare demographic data and pre-exercise variables with continuous and discrete scales, respectively, between the groups. The analyses indicated no significant differences between the groups for age (*t* = −0.85, *p* = 0.39), height (*t* = 0.34, *p* = 0.73), weight (*t* = −0.01, *p* = 0.99), BMI (*t* = −0.23, *p* = 0.99), years of education (*t* = 0.67, *p* = 0.50), resting HR (*t* = 0.85, *p* = 0.40), or MMSE score (*t* = 0.13, *p* = 0.90). Furthermore, no differences were observed for sex ratio (χ*^2^* < 0.91, *p* = 0.33). Descriptive data are summarized in Table 1.

### Exercise Manipulation Check

One-way repeated ANOVA was used to analyze HR in the exercise group, and the results revealed a significant time effect (*F*_(__3_,_32__)_ = 1027.75, *p* < 0.0001, partial η*^2^* = 0.99), indicating that the treatment HR was significantly higher than the immediately post-exercise HR and the follow-up HR, which was also significantly higher than the pre-exercise HR. The average RPE value during the resistance exercise was 15.25 ± 1.46. Descriptive data for the exercise manipulation check are summarized in Table 2.

**TABLE 2:** Descriptive data for the exercise manipulation check.

Variables	EG (M [SD])	CG (M [SD])
Pre-exercise HR (bpm)	73.66 [2.91]	72.86 [1.77]
Treatment HR (bpm)	122.74 [4.15]	73.86 [2.37]
Immediately post-exercise HR (bpm)	97.69 [2.76]	74.17 [2.04]
40-min post-exercise HR (bpm)	89.71 [4.06]	73.83 [3.15]
RPE	15.26 [1.46]	∼

*bpm, beats per minute; HR, heart rate; RPE, rating of perceived exertion.*

### Effects of Resistance Exercises on Stroop Performance

**FIGURE 1:**
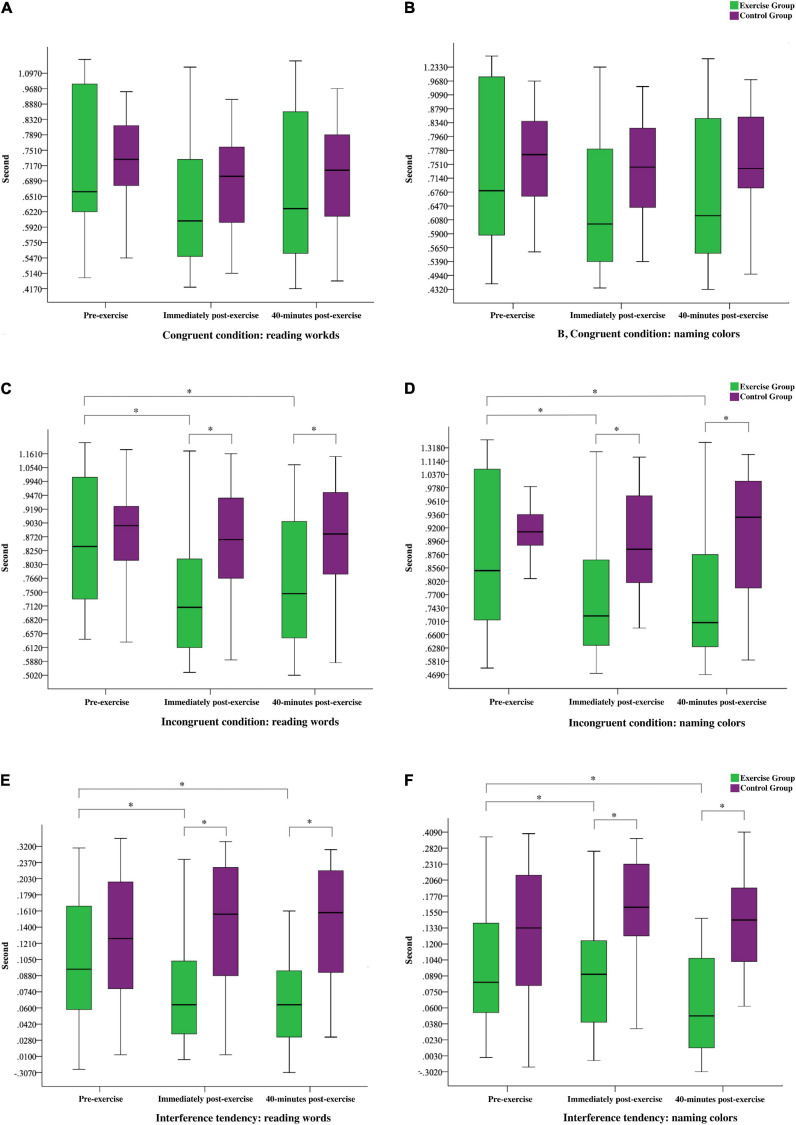
Stroop test performance on the pre-exercise, immediately post-exercise, and 40-min post-exercise; green indicates exercise group and purple indicates control group ^∗^ indicates a significant difference (*p* < 0.05) between the exercise and control groups or between pre-exercise, immediately post-exercise, and 40-min post-exercise.

In the congruent condition of reading words and naming colors, the 2 × 3 mixed ANOVA revealed a significant Time effect (*F*_(__2_,_67__)_ = 16.14 and 13.13, *p* < 0.0001, partial η*^2^* = 0.33 and 0.28) and significant interactions of Group with Time (*F*_(__2_,_67__)_ = 5.06 and 4.66, *p* < 0.01, partial η*^2^* = 0.13 and 0.12). However, no significant Group effect was observed (*F*_(__1_,_68__)_ = 0.41 and 0.01, *p* > 0.05). Follow-up decompositions indicated significant Time effects for the congruent condition of reading words and naming colors in the exercise group (*F*_(__2_,_33__)_ = 13.21 and 11.44, *p* < 0.001, partial η*^2^* = 0.45 and 0.41), wherein pairwise comparisons revealed that the participants exhibited faster reaction times (RTs) in the immediately and 40-min post-exercise tests than in the pre-exercise test. No RT difference was observed between the two post-exercise tests. As expected, no significant Time effects were observed for the congruent condition of reading words and naming colors in the control group (*F*_(__2_,_33__)_ = 2.96 and 1.92, *p* > 0.05). The results under the congruent condition are presented in Figures 1A,B.

To test the hypothesis regarding the effects of acute resistance exercise on the incongruent condition of reading words and naming colors, we conducted 2 × 3 mixed ANOVA. The results revealed significant Time effects (*F*_(__2_,_67__)_ = 17.06 and 13.80, *p* < 0.0001, partial η*^2^* = 0.34 and 0.29) and significant interactions of Group with Time (*F*_(__2_,_67__)_ = 9.67 and 10.88, *p* < 0.0001, partial η*^2^* = 0.22 and 0.25). The results also indicated a significant Group effect (*F*_(__1_,_68__)_ = 4.22 and 4.22, *p* < 0.05, partial η*^2^* = 0.06 and 0.06). The exercise group exhibited faster RTs than the control group did in the incongruent condition of reading words and naming colors immediately post-exercise and 40-min post-exercise (*t*_(__68__)_ = −3.04, −3.96, −3.02, and −3.07, *p* < 0.01). Follow-up decompositions indicated significant Time effects for the exercise group under the incongruent condition of reading words and naming colors (*F*_(__2_,_33__)_ = 20.72 and 20.64, *p* < 0.001, partial *η^2^* = 0.56 and 0.56), wherein pairwise comparisons revealed that the participants again exhibited faster RTs immediately and 40-min post-exercise than at pre-exercise, and no significant difference was noted between the post-exercise scores. As expected, no significant Time effects were observed for the incongruent condition of reading words and naming colors in the control group (*F*_(__2_,_33__)_ = 1.33 and 0.24, *p* > 0.05). The RTs under the incongruent condition are presented in Figures 1C,D.

To test the hypothesis regarding the effects of acute resistance exercise on the interference tendency of reading words and naming colors as indicators of inhibitory control, we conducted 2 × 3 mixed ANOVA. The results revealed significant Time effects (*F*_(__2_,_67__)_ = 5.04 and 3.49, *p* < 0.01, partial *η^2^* = 0.13 and 0.10) and significant interactions of Group with Time under the interference conditions (*F*_(__2_,_67__)_ = 6.36 and 4.01, *p* < 0.05, partial *η^2^* = 0.11 and 0.11). A significant Group effect was also observed (*F*_(__1_,_68__)_ = 26.07 and 47.19, *p* < 0.001, partial *η^2^* = 0.27 and 0.41). Notably, the exercise group had shorter RTs than the control group immediately and 40-min post-exercise (*t*_(__73__)_ = −5.17, −5.29, −5.04, and −6.07, *p* < 0.001). Follow-up decompositions indicated significant Time effects for the exercise group (*F*_(__2_,_33__)_ = 13.52 and 6.38, *p* < 0.01, partial *η^2^* = 0.45 and 0.28), wherein pairwise comparisons revealed that the participants exhibited faster RTs in the post-exercise tests than in the pre-exercise test; no significant RT difference was observed between the two post-exercise tests. As expected, no significant Time effects were observed for the control group (*F*_(__2_,_33__)_ = 1.17 and 0.08, *p* > 0.05). The results suggested that the responses of the exercise group were quicker both immediately post-exercise and 40-min post-exercise compared with those pre-exercise. They were also significantly quicker than those of the control group. The RTs for the interference tendency are presented in Figures 1E,F.

## Discussion

This study examined the effects of a bout of acute resistance exercise on inhibition performance in healthy middle-aged adults – specifically, the sustained effects of resistance exercise on the results of the Stroop test, which measures EF inhibition and attention. Exercise intensity was assessed using HR and RPE; HR was increased during the exercise session, immediately post-exercise, and 40 min post-exercise, reflecting physiological arousal. The participants in the exercise group had superior performance in RT under the interference condition and interference tendency immediately and 40 min post-exercise relative to the control group. These findings suggest that moderate-intensity resistance exercise has a beneficial effect on inhibition immediately post-exercise and 40 min post-exercise.

The findings are consistent with those of previous studies indicating that acute moderate-intensity resistance exercise has advantageous effects on the results of the congruent and incongruent conditions (Chang and Etnier,2009a,b; Tsai et al., 2014; Chang et al., 2019; Naderi et al., 2019). Given that neuroimaging techniques have indicated that the prefrontal cortex supports regulative control processes and is likely the neural substrate for the improved Stroop performance elicited by moderate resistance exercise (Chang Y. K. et al., 2017). One possible interpretation for the positive effects of acute resistance exercise on inhibitory control is that they might be related to the plasma levels of neurochemicals such as brain-derived neurotrophic factor and catecholamine (Ramel et al., 2004; French et al., 2007; McMorris et al., 2008). Findings regarding resistance exercise in middle-aged individuals suggest that such exercise could stimulate increases in HR and changes in catecholamine levels and that such changes might explain the general facilitation of performance under all Stroop test conditions associated with resistance exercise (French et al., 2007; Chang and Etnier,2009a,b). At the same time, resistance exercise may result in increased HR and may stimulate the secretion of catecholamine, with elevated levels of catecholamine being sustained 40 min after acute resistance exercise, which might, in turn, provide optimal force and energy supplementation.

However, the present hypothesis concerned whether the effect on inhibitory control would be sustained 40 min after the termination of acute resistance exercise. We observed an effect immediately after exercise, and the performance of the exercise group across a variety of the Stroop tasks supported the hypothesis of a sustained effect, because the beneficial effect in the exercise group compared with the control group was sustained for at least 40 min. The exercise group had high HR levels 40 min after the termination of exercise compared with the control group, although the arousal levels were much lower than those during and immediately after the exercise, suggesting that the mechanisms that mediate the immediate and sustained effects after the termination of exercise might explain the sustained effect. This finding could be explained by the cognitive-energetic model that the optimal arousal levels drive cognitive performance (Sanders, 1983). In our study, the intervention of acute resistance exercise induced an increase in HRs that may be related to the exercise-induced increase in arousal and lead to improved reaction time in inhibitory control. Furthermore, the inhibitory control performance was assessed immediately post-exercise while the heart rate was closed to moderate arousal level. Although, the exercise-induced arousal level would decrease with time, the effects were still sustained at 40-min post-exercise. Therefore, our study results supported and agreed with the cognitive-energetic model that resistance exercise has an immediate benefit on inhibition and the benefit can be sustained 40 min after the termination of acute exercise.

After the cessation of resistance exercise, the participants had higher levels of arousal immediately and 40 min post-exercise compared to the control group, while these arousal levels were significantly lower than those during exercise and immediately after the cessation of resistance exercise. Indeed, moderate exercise intensity may induce an optimal state of physiological arousal (e.g., heart rate) that is associated with facilitated inhibitory control performance (Kashihara et al., 2009). Although, the mechanisms could mediate the effects of resistance exercise immediately after the termination of resistance exercise. Nevertheless, previous studies by Chang Y. K. et al. (2017), Drollette et al. (2014), and Hillman et al. (2009) provided potential explanations for this discrepancy by using neuroelectric approaches. They have used event-related potentials (ERPs) to observe that cognition after acute exercise–induced arousal returned to within 10% of pre-exercise levels to avoid any general arousal effects on ERP measures stemming from exercise participation. Acute exercise elicited brain activation by increasing P3 amplitudes, shortening P3 latencies, or decreasing N450 latency during the performance of an executive control task. Their results suggested that attentional resource allocation and information processing efficiency during cognitive performance were promoted by acute exercise, moderate-intensity exercise may result in an optimal amount of resources. We designed the current study because examination of Stroop performance and exercise-induced arousal immediately post-exercise and 40 min post-exercise is necessary, and is to certain the relationship of the time course and resistance exercise to the benefit of inhibitory control. Protocols examining different time points provide information regarding the immediate effects of exercise on inhibitory control and indicate how long the effects continue; moreover, they reveal the role of arousal in the relationship between acute exercise and inhibitory control in middle age. Therefore, an active lifestyle has a protective effect on healthy brain function in older adults (Rolland et al., 2010). Acute resistance exercise is a potential preventive strategy that might benefit brain function by delaying the onset of cognitive decline and slowing brain disease progression; however, well-designed randomized controlled trials are required. Finally, with population aging, declines in EF performance among middle-aged healthy adults, associated with appropriate capacity for daily living skills, are of concern. Our findings support the benefits of resistance training for inhibitory control. Ascertaining appropriate prescriptions of moderate-intensity resistance exercise for maximizing these effects is desirable.

### Limitations and Future Research

This study had limitations that warrant caution with respect to the interpretation of its results and future research. Nonetheless, based on the findings of the present study, further research efforts in this field are suggested. First, the outcome of the study may have been affected by the small size and diversity of the sample; however, given the significance and size of the effects, we believe that a larger sample size would not have significantly altered the outcome. Second, an additional limitation of these data is the inability to gain a mechanistic understanding of the effects of acute resistance exercise on inhibitory control. That is, despite the interesting and positive sustained impacts about RT observed herein, little is known regarding which accuracy of interference condition and interference tendency on Stroop Test was influenced by an acute resistance exercise bout. Third, future studies should be designed to further our understanding of the measurement of intra-subject variation (i.g., standard deviation and coefficient of variation) for RTs. Meanwhile, the recording of ERPs could be used to explore stimulus and response conflicts within the processes underlying standard deviation and coefficient of variation for RTs. Whether standard deviation and coefficient of variation for RTs are related to localized brain regions is unknown, and neuroimaging could provide clarification. Because the intra-subject variation in RTs is a measure of a subject’s consistency in responding to the conditions of congruent and incongruent stimuli, often quantified as the standard deviation and coefficient of variation of intra-subject variation across a task period; higher intra-subject variation, reflect in larger standard deviations, is associated with greater variability, or inconsistency, of responses. Finally, resistance exercise training and arousal-induced alterations in lateral prefrontal cortex neural activity (Yanagisawa et al., 2010), biochemical release (Hillman et al., 2009; Chang et al., 2014), and cerebral blood flow (Chang H. et al., 2017) have been posited. Future investigations regarding the relationships between resistance exercise, cognitive performance, and biophysiological mechanisms in middle-aged adults are necessary.

## Conclusion

Our findings suggest that moderate-intensity resistance exercise promotes EF in healthy middle-aged adults. In summary, this study has extended the literature by providing evidence that acute resistance exercise has positive, sustained impacts on multiple cognitive functions, as assessed by the Stroop test, in healthy middle-aged adults. Furthermore, the results suggest that moderate-intensity resistance exercises have positive impacts and sustained effects on particular types of EF in middle-aged adults, with those impacts being larger under task conditions that place greater demands on inhibitory control cognition.

## Data Availability Statement

The raw data supporting the conclusions of this article will be made available by the authors, without undue reservation.

## Ethics Statement

The studies involving human participants were reviewed and approved by the University of Taipei’s Institutional Review Board (IRB-2020-011). The patients/participants provided their written informed consent to participate in this study.

## Author Contributions

C-CC and C-JH contributed to conception and design of the study and wrote sections of the manuscript. M-CH and Y-HC organized the database. W-YW and M-YH performed the statistical analysis. C-CC, M-CH, Y-HC, W-YW, MY-H, and C-JH wrote the first draft of the manuscript. All authors contributed to manuscript revision, read, and approved the submitted version.

## Conflict of Interest

The authors declare that the research was conducted in the absence of any commercial or financial relationships that could be construed as a potential conflict of interest.

## Publisher’s Note

All claims expressed in this article are solely those of the authors and do not necessarily represent those of their affiliated organizations, or those of the publisher, the editors and the reviewers. Any product that may be evaluated in this article, or claim that may be made by its manufacturer, is not guaranteed or endorsed by the publisher.
